# Cemented and screw-retained implant-supported single-tooth restorations in the molar mandibular region: A retrospective comparison study after an observation period of 1 to 4 years

**DOI:** 10.4317/jced.51708

**Published:** 2015-02-01

**Authors:** Alberto Ferreiroa, Miguel Peñarrocha-Diago, Guillermo Pradíes, María-Fernanda Sola-Ruiz, Rubén Agustín-Panadero

**Affiliations:** 1 DDS, PhD, Associate Professor of the Department of Buccofacial Prostheses. Faculty of Odontology. Complutense University of Madrid, Madrid, Spain; 2 DDS, PhD, Chairman of Oral Surgery. Director of the Master in Oral Surgery and Implantology. Valencia University Medical and Dental School. Valencia, Spain. Investigator of the IDIBELL Institute; 3 DDS, PhD, Professor and Director of the Department of Buccofacial Prostheses. Faculty of Odontology. Complutense University of Madrid, Madrid, Spain; 4 DDS, PhD, Adjunct Professor of the Occlusion and Prosthodontic Teaching Unit. Department of Stomatology. Valencia University Medical and Dental School. Valencia, Spain; 5 DDS, PhD, Associate Professor of the Occlusion and Prosthodontic Teaching Unit. Department of Stomatology. Valencia University Medical and Dental School. Valencia, Spain

## Abstract

Objectives: The aim of this study was to evaluate the survival and compare the appearance of different mechanical and biological complications, in screw-retained and cemented-retained single-tooth implant-supported restorations localized in the molar mandibular region, over a period of 1 to 4 years. Material and Methods: A retrospective study was carried out with a total of eighty implant-supported restorations, which were placed in eighty patients for prosthetic rehabilitation of a mandibular molar. Forty patients were rehabilitated with a cemented-retained restoration and the other forty with a screw-retained restoration. The presence of the following complications was recorded for both types of prostheses: Fractures of the ceramic veneering, loosening screws, mucositis and peri-implantitis. Debonding of the restoration was analyzed in the cemented-retained restoration group. The clinical survival of crowns was analyzed with a Kaplan-Meier test and the clinical complications were compared, using a Student t test and Log-rank test. Results: 27 patients registered some complication. The average rate of complications was 37,5% for cemented-retained restorations and 30% for screw-retained restorations. The complications more common in the cemented-retained restoration were the presence of mucositis (14,87%), while in the screw-retained restorations was the loosening screw (20%). Student t test and Log-Rank test found significant differences (p=0,001) between the screw loosening and presence of mucositis. Conclusions: The cemented-retained restorations seem to prevent screw loosening, but the presence of cement seem to increase the complications around the soft tissues, however in the screw-retained restorations the presence of mucositis and peri-implantitis are lower than cemented-retained restorations. The incidence of fracture of ceramic veneering was similar in both groups.

** Key words:**Screw-retained restorations, cemented-retained restorations, screw loosening, peri-implant diseases and fracture ceramic veneering.

## Introduction

Single-tooth replacements with osseointegrated implants in the posterior mandibular sector has become a routine treatment instead of fixed dental restorations, showing good long-term survival results (1). At present, the prosthodontic rehabilitation of implants can be performed through a cemented or screw-retained restoration. The election of the prostheses is still usually based on the preferences of the clinicians (2), having many clinical situations, where both types of prostheses can be used interchangeably. Nevertheless, different advantages and disadvantages have been described for each type (3).

In the case of cemented restorations different types of advantages have been described in the scientific literature (4,5). They have a good esthetic appearance due to absence of the occlusal hole (3,6). A correct passive fit can be obtained with these restorations, because the layers of the luting agent compensate the misfit between abutment and restorations (7). Also, the cemented restorations seem to have a better fracture resistance of the ceramic veneering than screw-retained restorations (8,9). As for disadvantages, we need an adequate prosthetic space and in several cases we can have difficulties in the removal of the prostheses (10). In an effort for simplicity a possible retraivibility for these cemented crowns the use of temporary luting agent has been proposed for the cementing (11). Nevertheless, several studies revealed (12-14), that the temporary luting agents, as polyurethane agents, obtained similar retention results as permanent cement, as for example zinc phosphate cement in single-tooth implant-supported restorations, so that in many cases is necessary to destroy the entire restoration, when the removal the crown is required. Moreover, after the cementation procedure excess removal of the luting agent used can be difficult and the presence of residual cement can be a risk factor of mucositis and peri-implantitis (15). Several studies (15-18) have reported different biological complications, as peri-implant inflammation, soft-tissue swelling, bleeding, and loss of crestal bone with these types of prostheses.

Many clinicians prefer the screw-retained restorations, because we can avoid the presence of residual cement, decreasing the complications in the soft tissue (19,20). Furthermore, the clinicians can easily remove these res-torations, when repairing of the ceramic veneering is necessary. Nevertheless, the hole for introducing the screw in the occlusal face of the crown produces a non-esthetic restoration and can disrupt the normal occlusal contacts (3,6,21). Additionally, the fracture resistance of the porcelain can be lower than cemented restorations, due to the presence of the screw access. Moreover, this screw access can produce the appearance of shear-flexural stresses in the occlusal third of the screw, with an increase of cohesive and adhesive failure in the bond between ceramic and framework (22).

At present, the implant systems available offer various prosthetic solution for cemented and screw-retained single-tooth restorations, however very little scientific information is available, comparing the pros and cons of both type of prostheses in similar conditions, there is not enough scientific evidence that one type of restoration is superior to the other. The purpose of this prospective study was to analyze and compare the presence of screw loosening, fracture of ceramic veneering, mucositis and peri-implantitis and the survival rate of cemented and screw-retained single-tooth posterior restorations in similar conditions. At the same time, the debonding of the crown was analyzed for cemented-retained restorations.

## Material and Methods

98 patients were rehabilitated with one implant on the mandible in a private implant center, in the molar mandibular region, between January 2008 to December 2012. The inclusion criteria for rehabilitating this patients, were the following: a] Enough prosthetic space; at least 8 mm from the level of the soft tissues to the cusp of the antagonist molar, b] Occlusal scheme allowing for the establishment of correct occlusal cuspfosse contacts, c] Patients with an adequate disponibility, for going to the revision appointments, d] No previous history of periodontitis and, e] Presence of distal molar tooth, f] Time minimum of 1 year follow-up.

For the surgical phase, an external connection implant [Lifecore Restore 4,1 mm RD, Lifecore Biomedical Inc, Chaska MN, USA] was used. The same practitioner in one-stage surgery placed all implants. 3 months after the placement of the implants, the prosthetic phase was begun. In the prosthetic phase, all prostheses were done by an experienced prosthodontist, who decided the type of prosthesis for each patient. Forty patients were restored with cemented restorations and the others forty with screw restorations For the impression, a conventional tray was used to do a closed tray technique to take an impression with elastomeric material [Impregum, 3M-espe, St.Paul MN, USA]. Afterwards, an artificial stone type IV [FujiRock, Gc Corporation, Tokyo, Japan] and a gingival mask [Gimask automix, Coltene/whaledent, Altstätten, Switzerland] were used for pouring the impression.

The same lab technician fabricated all of the restorations. For cemented-retained restorations, a prefabricated abutment [COC Abutments, Lifecore Biomedical Inc], which was milled in function of the individual features of implant angulation and peri-implant mucosal contour, and a metal-ceramic crown, which was manufactured with a Co-Cr metal-alloy [Remaniun Co-Cr alloy Co 61%, Cr 25%, Mo 7%, W 5%, Si 1.5%, Mn, N < 1%, Dentaurum, Ispringen, Germany] and a feldspathic ceramic veneering [IPS d.SIGN, Ivoclar Vivadent, Schaan, Liechtenstein], were used. The access hole of the prefabricated abutment was closed with a teflon pellet and later the crowns were cemented with non-eugenol temporary cement for implant-retained crowns [Premier Implant cement, premier dental products, Plymouth Meeting PA, USA].

For screw-retained restorations an UCLA castable abutment [UCLA gold/plastic combo sleeve, Lifecore Biomedical Inc] was used. The patterns of the structures of the screw-retained restorations were individually customized by applying modeling wax on the UCLA abutment. Posteriorly, the patterns were invested in a commercial phosphate-bonded investment and the vacuum casting of the structures was done with an induction centrifugal machine, under a pressure of 580 mm and a temperature of 1465 ºC. The access hole of the screw-retained crowns was closed with a teflon pellet and a hybrid resin composite [Tetric-Ceram, Ivoclar Vivadent, Schaan, Liechtenstein]. All screws for both types of prostheses were tightened with a torque of 30 Ncm according to the manufacturer’s specifications. On the day the crown was placed, the patients were instructed in oral hygiene techniques for a correct maintenance.

After the prosthetic phase, the revisions of the patients were done every 12 months. A clinical inspection was done to register signs of mucositis or peri-implantitis, and presence of fracture of ceramic veneering, loosening screw and debonding in cemented-retained crowns. The parameter used for the diagnosis of mucositis is bleeding on gentle probing [0,25 N/cm2], while for the diagnosis of peri-implantitis, changes in the level of the crestal bone in conjunction with bleeding on probing with or without concomitant deepening of peri-implant pockets and presence of pus were the parameters used for the diagnosis of peri-implantitis (23). In all patients with anterior signs, a radiographic control was done to evaluate the marginal bone loss using a periapical radiographic, which were taken using a paralleling technique with a ring holder [Super-Bite Posterior, Kerr Dental, Orange CA, USA]

Statistical analysis was done, using SPSS version 15.0 for Windows [Microsoft Inc, Redmond WA, USA]. A paired Student t test was used to evaluate the presence of screw loosening, fracture of the ceramic veneering and presence of mucositis or peri-implantitis, comparing cemented and screw-retained restorations. Moreover, ove-rall survival was obtained by Kaplan-Meier survival curves and compared with Log-rank test. The significance level was set at *P* < 0.05.

## Results

**Figure 1:**
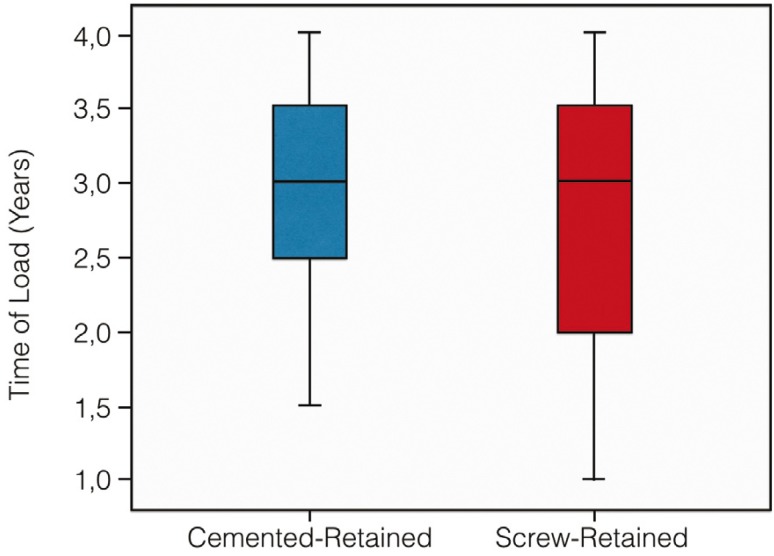
Boxplot with the distributions of the load time in both type of prostheses.

18 patients were excluded in this study; because of there was no follow-up 1-year, so that 80 patients [42 women and 38 men] with a mean age of 44.4 years were included in the study. In one patient, the implant failed during the osseointegration period, after three months a new implant was placed and this patient was included in the study. Time load of both types of prostheses was homogeneous; there were no statistically significant differences in the mean time observation of cemented and screw-retained restorations [*p*=0,599] (Fig. 1).

A total of 27 patients had some type of complication [15 and 12 patients for cemented and screw-retained restorations, respectively]. The average rate of complications for cemented-retained restorations was 37,5% ± 7,7% and 30% ± 7,3% for the screw-retained restorations ( Table 1). For cemented-retained restorations group the mechanical complications were the screw loosening, debonding of the crown and cohesive fracture of the veneering for 2, 5, 2 patients respectively. In the case of screw-retained restorations the screw loosening was registered in 8 patients and cohesive fracture of the ceramic veneering in 4 patients. In the case of mucositis or peri-implantitis, 7 patients in the group of cemented-retained restorations showed signs of mucositis, and 1 patient showed signs of peri-implantitis with a crestal bone loss of 1,5 mm after 36 months. In the screw-retained restorations group only 2 patients showed mucositis signs. In the radiographic control, all cases of the cemented-retained restorations group with complications in the soft tissues showed cement remnants.

**Table 1:**
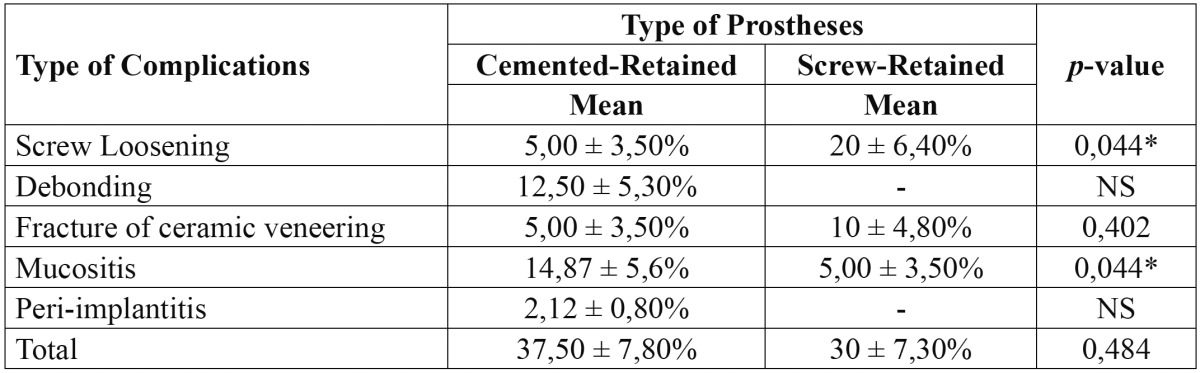
Rate of clinical complications.

A paired Student t test detected statistically significant differences, when comparing the screw loosening [*p*=0,044*], however did not detect significant differences, when comparing the fracture of the veneering. In contrast, when comparing the presence of mucositis, the Student t test detected significant differences [*p*=0,044*]. The debonding of the cemented crowns was not compared, because of is an exclusively complication of the cemented prostheses. In the same way, we only obtained incidence of peri-implantitis with cemented-retained restorations, so that this complication was not statistically compared ( Table 1).

**Figure 2:**
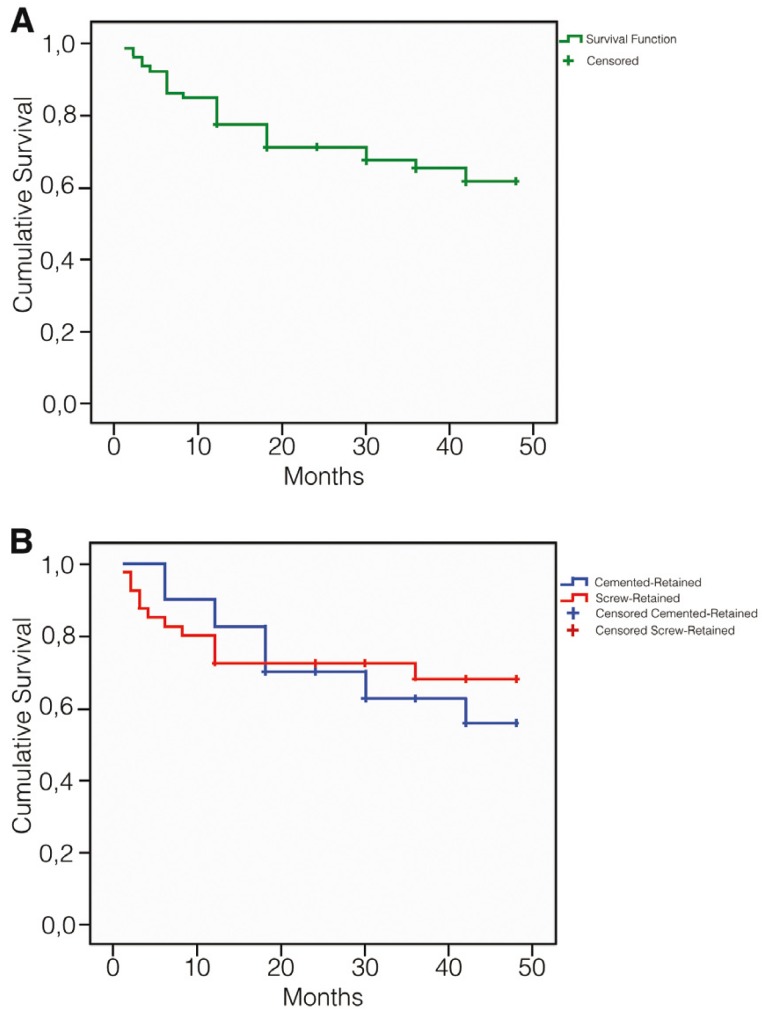
Overall survival curve. A) Of all restorations of the study; B) Of cemented-retained and screw-retained restorations, without differences.

**Figure 3:**
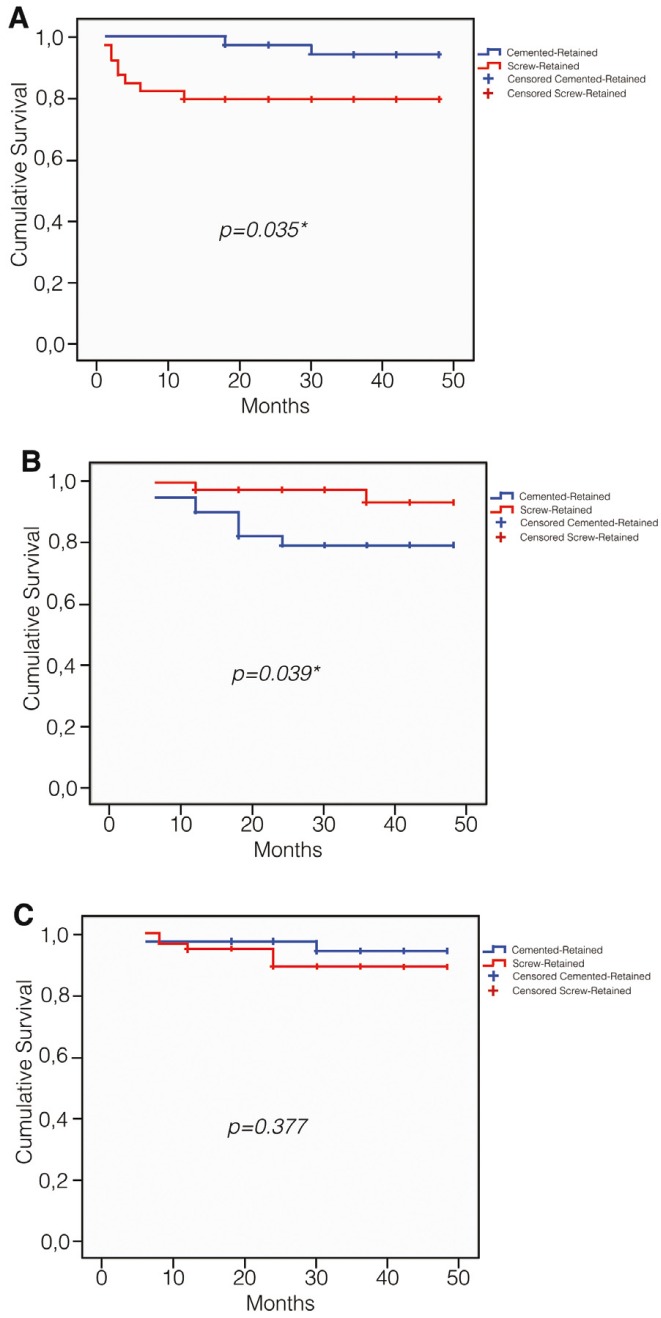
Overall survival curve. A) Of screw loosening; B) Of presence of mucositis; C) Of fracture of the ceramic veneering.

The clinical survival rate of the 80 restorations of the study with any type of complication was 38,3%, and the comparison of Kaplan-Meier survival curves of both type of prosthesis showed not significance differences with the Log-rank test (Fig. 2). When we compare the Kaplan-Meier survival curves for screw loosening, fracture of the ceramic veneering and presence of mucositis, the Log-rank test detected significance differences for screw loosening [*p*=0,035] (Fig. 3) and presence of mucositis [*p*=0,039] (Fig. 3), but not for the fracture of the ceramic veneering [*p*=0,377] (Fig. 3), in accordance with Student t test.

## Discussion

In this Retrospective study the Student t test and Log-rank test did not detect significant differences in the apparition of total complications in both types of single-tooth implant-supported restorations of this study. Nevertheless the statistical analysis found differences in the screw loosening. The abutment screw loosening is a frequent complication in implant-supported restorations with a range between 3% and 45% (24) of apparition, in our case this type of complication was more common in screw-retained restorations with 20% than cemented restorations with 5%. The rate of apparition of this type of complication is similar to the data, which there are in other clinical studies in the scientific literature (25-27).

Moreover, when we analyzed the soft tissues complications, we found differences between two groups of restorations. The soft tissue complications in dental implants have been associated to dental cements (15,16,28-30). Several studies (15,29,30) found a correlation of mucositis and peri-implantitis with residual cement in the soft tissues. In our study 7 patients of the cemented-retained crowns group showed presence of mucositis and 1 patient with peri-implantitis, all these patients had presence of residual cement around soft tissues. When the incidence of symptoms of mucositis was evaluated in the screw-retained crowns groups only two patients registered evidence of this pathology, showing this group to have a lower incidence of this type of complication. In this clinical study the peri-implant soft tissue responded better with screw-retained restorations than cemented-retained restorations, so the excess of the cement in implant-supported restorations may have a similar behavior than calculus, favoring the development of the mucositis and peri-implantitis. Linkevicius *et al.* (30) found in 73 implants restored with cemented-retained restorations, evidences of residual cement. Within these implants, 34 were placed in patients without history of periodontitis, 20 showed mucositis and 3 early peri-implantitis and other 39 implants were placed in patients with history of periodontitis, obtaining 35 implants with peri-implantitis and 3 with early peri-implantitis. In our study 8 implants showed signs of complications in the soft tissues and in all cases residual cement were found in the radiographic exam. Our incidence of these complications is lower than Linkevicius *et al.* (30), because of we did not include patients with previous history of periodontitis, however the presence of the cement seem to be a predisposing factor for these type of complications, so that in cemented-retained restorations, the removal of the residual cement is a priority for decreasing the incidence of mucositis and peri-implantitis.

On the other hand, we have not found statistical differences in the fracture of the ceramic veneering. This type of complication was similar in both types of prosthesis, however several studies (9,22,30) reported that the screw access in the screw-retained restorations can weaken the ceramic veneering and produce a fracture, this screw access cuts off the structural continuity of porcelain, modifying the position of the centre of the mass of the ceramic bulk. Zarone *et al.* (22) reported in an in Vitro, that the fracture resistance of ceramic veneering in cemented-retained restorations [1657 N/cm2] is higher than the screw-retained restorations [1281 N/cm2]. Al-Omari *et al.* (9) reported similar results, with higher results in cemented-retained restorations [3707 N/cm2] versus screw-retained restorations [1885 N/cm2]. Nevertheless, the maximum bite force in the region of the first molar is from 330 N/cm2 to 880 N/cm2 (31). The different in Vitro Studies (9,22,30,31) reported resistance values higher than maximum bite force in this area for both types of prosthesis, so the rate of the fracture of the ceramic veneering should be similar, as in our case.

Debonding of the cemented-retained crowns is a complication, which was only analyzed in these restorations. In our study, we used a polyurethane-luting agent, which is included in the group of semipermanent or provisional cement (32). Schwarz *et al.* (14) in a clinical study used two types of provisional cement with similar loss retention than permanent cement in single crowns. These authors reported the loss of retention occurred in a 11,6% in the single crowns, with a similar result obtained for us [12,5%].

Results of the present clinical study seem to indicate that the cemented restorations could prevent loosening problems, but the incidence of mucositis and peri-implantitis are more likely. In the screw restorations, the behavior seems to be exactly the opposite, with more incidence of loosening screw, but fewer incidences of mucositis and peri-implantitis. Nevertheless, both types of prostheses can be perfectly valid for solving these types of cases for the restoration a single implant in the mandibular molar region.

## Conclusions

Within the limits of the present clinical study, the following conclusions can be drawn:

1. The cemented-retained restorations show lower incidence of screw loosening than screw-retained restorations.

2. The complications of the soft tissues showed statistically significant results, so the type of restoration used influences the frequency of occurrence of these complications.

3. The screw hole-access did not affect the fracture resistance of the ceramic veneering in the screw-retained restorations.

4. All 80 implants of this clinical study survived, so that both type of prosthetic options may be valid for these type of implant restorations in this region.
